# Eradicating latent tuberculosis: use of interferon gamma release assay and isoniazid/rifapentine in people living with HIV/AIDS

**DOI:** 10.1590/S1678-9946202567014

**Published:** 2025-03-03

**Authors:** Mariana Amélia Monteiro, Carlos Fernando Apoliano, José Eduardo Rodrigues Martins, Noemia Orii Sunada, Víctor Ângelo Folgosi, Najara Ataíde de Lima Nascimento, Erica Chimara, Ana Paula Rocha Veiga, Luisa de Oliveira Pereira, Luisa Caracik de Camargo Andrade, Larissa Tiberto, Maurício Domingues Ferreira, Luiz Augusto Marcondes Fonseca, Alberto José da Silva Duarte, Denise Arakaki-Sanchez, Marisa Ailin Hong, Jorge Casseb

**Affiliations:** 1 Universidade de São Paulo, Faculdade de Medicina, Hospital das Clínicas, Departamento de Dermatologia, Ambulatório de Imunodeficiências Secundárias, São Paulo, São Paulo, Brazil; 2 Universidade de São Paulo, Faculdade de Medicina, Departamento de Dermatologia, Laboratório de Imunodeficiências Secundárias (LIM-56), São Paulo, São Paulo, Brazil; 3 Instituto Adolfo Lutz, São Paulo, São Paulo, Brazil; 4 Ministério da Saúde, Secretaria de Vigilância em Saúde e Ambiente, Departamento de HIV/Aids, Tuberculose, Hepatites Virais e Infecções Sexualmente Transmissíveis, Brasília, Distrito Federal, Brazil; 5 Secretaria de Estado da Saúde do Distrito Federal, Brasília, Distrito Federal, Brazil; 6 Universidade de São Paulo, Faculdade de Medicina, Instituto de Medicina Tropical de São Paulo, São Paulo, São Paulo, Brazil

**Keywords:** Tuberculosis, Isoniazid, Rifapentine, 3HP, Chemoprophylaxis, Brazil, Incidence

## Abstract

Tuberculosis (TB) is the most common comorbidity in people living with HIV/AIDS (PLWH), including those under antiretroviral treatment. PLWH are 28 times more likely to develop TB in Brazil, the leading cause of HIV-related deaths globally, with approximately 161,000 reported deaths worldwide in 2023. Early diagnosis of latent tuberculosis infection (LTBI) and prophylactic therapy can reduce TB cases, prevent disease progression, and decrease transmission in high-risk populations. This study assessed the prevalence of LTBI in PLWH using the interferon-gamma release assay (IGRA) and the impact of the 3HP regimen (isoniazid [INH]/rifapentine [RPT]) as prophylactic treatment. Blood samples from 335 PLWH (78% of the 427 in the cohort) were tested for IGRA; 50 PLWH (15%) tested positive and were treated with 3HP. Treatment included 900 mg of INH and 900 mg of RPT in 12 weekly doses according to the Brazilian health guidelines. No specific risk factors, including nadir CD4+T count, age, gender, or antiretroviral therapy (ART), were more frequently observed in the PLWH with LTBI compared to the PLWH without LTBI. All PLWH with LTBI received treatment and no cases of active TB were observed. Our findings highlight the need for wider LTBI screening and treatment among PLWH in the latent phase, emphasizing more stringent approaches for implementing 3HP prophylaxis.

## INTRODUCTION

Global tuberculosis (TB) statistics for 2023 from the World Health Organization (WHO) reveal a significant public health challenge. It is estimated that a quarter of the world’s population is infected with *Mycobacterium tuberculosis*, with 10.8 million new cases reported globally, resulting in 134 cases per 100,000 individuals^1^. Furthermore, people living with HIV/AIDS (PLWH) are 14–18 times more likely to develop TB compared to those without HIV^1,2^. The risk is even higher in developing countries, where PLWH are 20–30 times more likely to develop TB. In Brazil, e.g., this risk was 28 times higher in 2018^3^. In addition, WHO estimates that PLWH accounted for about 6.1% of all new TB cases, with approximately 658,800 new cases in this population in 2023^1^. TB remains the leading cause of death among PLWH, responsible for 161,000 deaths (13% of all TB-related deaths), highlighting the critical need for prevention and treatment strategies in this vulnerable group^1-3^.

Most individuals with latent tuberculosis infection (LTBI) remain asymptomatic for their whole life and are unaware of their condition. However, 5 to 10% of the global population, as well as PLWH, may progress to active disease, most commonly within two to five years after infection^1,4,5^. WHO suggests the identification and prophylactic treatment of LTBI as an effective strategy for reducing the transmission, morbidity, and mortality of active tuberculosis disease^6^. In a previously published article, we demonstrated that TB remains a leading cause of morbidity and mortality in PLWH in Sao Paulo, despite the effectiveness of combined antiretroviral therapy (cART)^7^. Therefore, TB prophylaxis via detection and treatment of LTBI, especially in high-risk populations, are important public health measures to eliminate or reduce the cases in PLWH.

The interferon-gamma release assays (IGRAs) quantifies (in vitro) the cellular immune response of T lymphocytes to specific *M. tuberculosis* antigens by detecting the interferon-gamma cytokine (IFN-γ) released by these cells. These tests use a combination of peptides mycobacterium ESAT-6 and CFP-10 to activate cells in vitro. These proteins are absent in all BCG strains and most nontuberculous mycobacteria, with a few exceptions like *M. kansasii*, *M. szulgai*, *M. marinum*, *M. ulcerans*, *M. haemophilum*, and species of the *Mycobacterium abscessus* complex^8^. Two IGRAs have been approved by the Food and Drug Administration (FDA) and are currently available in the USA: the QuantiFERON-TB Gold Plus (Qiagen, Hilden, Germany) and the T-SPOT.TB (Euroimmun, Lübeck, Deutschland) assay. IGRAs offer some advantages over the Tuberculin skin test (TST) including higher specificity, as less cross-reactivity with nontuberculous mycobacteria and the BCG vaccine^9-11^.

According to current Brazilian guidelines, PLWH are tested for LTBI at the time of HIV diagnosis, and annually if the IGRA test result is negative^11^. The 3HP regimen, which combines isoniazid (INH) and rifapentine (RPT), managed weekly for three months, is an alternative to the traditional six to nine months isoniazid regimen and has been recommended for adults and children by the Centers for Disease Control and Prevention (CDC) and WHO^1^. This study aimed to evaluate the prevalence of LTBI in PLWH using IGRA and to assess the impact of the 3HP regimen as a prophylactic therapy.

## MATERIALS AND METHODS

The pioneering outpatient service, established in 1983 as a branch of the Clinical Immunology Service at the Hospital das Clinicas of the Faculdade de Medicina of the Universidade de Sao Paulo, initially included approximately 600 PLWH, of whom 45 (8%) died during follow-up, mostly due to noninfectious causes^12^. Currently, 427 of them remain under active follow-up. For this study, we included individuals from January 2021 to December 2024, when IGRA was available in the public service in Sao Paulo city. During clinical follow-up, PLWH were invited to participate in this research to identify LTBI, regardless of gender, race, clinical and laboratorial stage. The exclusion criteria were those with previous tuberculosis, pregnant women, and other conditions listed by the Ministry of Health Guideline^11^.

Of the 427 individuals under active follow-up, 335 (78%) PLWH participated in our study and were tested by IGRA. Blood samples were collected in heparin tubes and then transferred to the QuantiFERON^®^-TB Gold Plus kit (Qiagen, Hilden, Germany), following the manufacturer’s protocol for the assay.

All people living with HIV who tested positive for latent tuberculosis infection were treated with the 3HP regimen (isoniazid [INH] and rifapentine [RPT]) in 12 weekly doses, following Brazilian health guidelines for TB management among PLWH^11^. Before starting LTBI treatment, active TB was ruled out via clinical evaluation, as the LTBI treatment should not commence if active TB is suspected. This included questioning about symptoms such as cough, fever, weight loss, and night sweats, as well as sputum examination (if there was a productive cough) and chest X-ray (even if asymptomatic). During clinical follow-up, laboratory tests, including liver function tests, were performed if there was an increased risk of hepatotoxicity, such as in alcoholics, or when clinically indicated.

Epidemiological, clinical, and laboratory data were obtained from PLWH via interviews during their follow-up and/or from their medical records. The study was approved by the Ethics Committee of Universidade de Sao Paulo (process Nº 0211/2010-TB MS).

### Statistical analysis

The parametric Student’s t-test was used to compare continuous variables with equal variances, nonparametric Mann-Whitney’s test for different variances, Fisher’s test and Chi- squared were used for categorical variables. The variables analyzed for risk factors to LTBI include: proportion of gender (men and women) and ethnicity (White, Black, and Mixed-race [including Indigenous individuals]), mean age, CD4+ T-Cells, Nadir CD4+ T-Cells, Zenith CD4+ T-Cells, mean and min/max CD8+ T-Cells, CD4/CD8 ratio, HIV-1 Viral Load, AIDS status before or during IGRA, and the time of cART.

## RESULTS

A total of 427 PLWH were monitored in our outpatient service from December 2021 to September 2024. Among them, 335 (78%) were tested by IGRA and50 (15%) tested positive for LTBI. Of this group, 38 (76%) were men and 12 (24%) were women (p=0.6). No significant differences were observed in the homogeneous representation among both men (p=0.554) and women (p=0.727).

The mean age of PLWH with LTBI was 52 years, compared to 53 years for those who tested negative (p=0.4). The mean CD4+ T cell count was 907 cells/mm^3^ in positive cases and 823 cells/mm^3^ in negative cases (p=0.12). Similarly, the mean CD4/CD8 ratio was 1.2 in the LTBI-positive group and 1.0 in the negative group (p=0.19). Furthermore, 88% of PLWH with LTBI had an undetectable HIV-1 viral load, compared to 89% of those without LTBI (p=0.45). No specific risk factors, including nadir T CD4+ count, age, gender, or ART, were more frequently observed in the PLWH with LTBI group compared to the PLWH without LTBI group (Table 1).

**Table 1:** Interferon gamma release assay (IGRA) for latent tuberculosis infection among 335 people living with HIV-1, from December 2021 to September 2024.

Parameter Quantiferon-TB	Positive	Negative	P value
Number tested (335, 100%)	50 (15%)	285 (85%)	
Sex/Ethnicity [n(%)]			0.610
Males	38 (76%)	205 (72%)	
White	27 (71%)	139 (68%)	
Black	5 (13%)	23 (11%)	0.554
Mixed-race	6 (16%)	43 (21%)	
			
Females	12 (24%)	80 (28%)	
White	7 (58%)	53 (66%)	
Black	2 (17%)	8 (10%)	0.727
Mixed-race	3 (25%)	19 (24%)	
Age [mean (± SD)]	52 (12)	53 (11)	0.404
CD4+ T-Cells [mean cells/mm^3^ (± SD)]	907 (338)	823 (354)	
CD4+ T-Cell [min/max cells/mm^3^]	48/1859	89/2289	0.122
Nadir CD4+ T-Cells [mean cells/mm^3^ (± SD)]	358 (228)	327 (201)	
Nadir CD4+ T-Cells [min/max cells/mm^3^]	26/992	5/1556	0.336
Zenith CD4+ T-Cells [mean cells/mm^3^, (± SD)]	1031 (322)	966 (394)	
Zenith CD4+ T-Cells [min/max cells/mm^3^]	317/1704	110/2729	0.205
CD8+ T-Cells [mean cells/mm^3^, (± SD)]	871 (382)	851 (386)	
CD8+ T-Cells [min/max cells/mm^3^]	295/2200	112/2329	0.728
CD4/CD8 ratio [mean (± SD)]	1.2 (0.6)	1.0 (0.5)	
CD4/CD8 ratio [min/max]	0.07/2.67	0.04/4.1	0.192
CD4/CD8 ≥1 [n (%)]	31 (62%)	133 (47%)	
CD4/CD8 <1 [n (%)]	18 (36%)	144 (50%)	
HIV-1 Viral Load undetectable <20 copies/mL [n (%)]	44 (88%)	254 (89%)	0.448
AIDS (CD4+ T-Cells <350 Cell/mm^3^) before or during IGRA [n (%)]	31 (62%)	181 (63%)	0.874
Time of cART in months [mean]	139	144	0.679

Statistical analysis = Student’s *t*-test unpaired parametric, nonparametric Mann-Whitney’s test, Fisher’s exact test, Chi-squared test; Nadir T-CD4 cells/mm^3^ = the lowest T-cells count lifetime; Zenith = the highest T-CD4 cells/mm^3^ lifetime; cART = Combined antiretroviraltherapy.

Those who tested positive for IGRA were treated with the 3HP regimen, consisting of 900 mg of isoniazid (INH) and 900 mg of rifapentine (RPT) managed in 12 weekly doses, in accordance with the Brazilian health guidelines. No cases of active TB were identified in PLWH who were tested by IGRA throughout the 34 months (2021-2024) of this study, in which patients were tested once in one to three years in their clinical laboratory routine tests, but in contrast, three untreated PLWH died from active TB before the implementation of this strategy. Additionally, we had three (0.9%) cases with an indeterminate result by IGRA. These cases underwent medical evaluation to decide on retesting. Unless patients have apparent symptoms that warrant immediate retesting, which were not observed, they will be re-evaluated during their next routine tests.

## DISCUSSION

From the 427 currently in active follow-up during this study, we evaluated 335 PLWH by IGRA, of whom 15% were positive to LTBI, but the prevalence in other regions and countries can vary^13^. PLWH with TB impose a higher economic burden on the health system than HIV/AIDS alone and LTBI/HIV, indicating that preventive TB treatment can avoid further costs of treating active TB^14^. In developed countries, such as Switzerland, LTBI prevalence declined from 15.1% in 2001 to 4.6% in 2021, and TB incidence declined from 90.8 cases/1,000 person-years in 1989 to 0.1 in 2021^15^.

In our statistical analysis, no specific risk factors were more frequent regardless of LTBI. This included variables such as Nadir CD4+ T cell count, age, gender, ethnicity, viral load, AIDS, and antiretroviral therapy (ART) status. Following the implementation of our screening and treatment strategy, all PLWH who were diagnosed with LTBI received appropriate treatment. Remarkably, we did not observe any cases of active TB in our cohort after the onset of this strategy. This highlights the effectiveness of the screening and treatment approach in preventing the progression from LTBI to active TB in this high-risk population.

Risk factors that are associated with IGRA positivity, such as immunosuppression characterized by lower CD4+ T cell counts, advanced age, and inadequate antiretroviral therapy adherence, underscore the importance of targeted screening in high-risk populations. The response to IGRA in PLWH with CD4+ T cell counts <350 cells/mm^3^ is particularly relevant^16^, as such patients may not recover immune competence even with antiretroviral control of HIV replication and may have a higher risk of developing tuberculosis over their lifetime.

Our cohort demonstrates a high level of adherence to cART, with over 95% of patients taking their medication consistently, which may not be representative of other regions in Brazil. This strong adherence is reflected in the high rate of PLWH with undetectable viral load (<20 copies/mL), exceeding 95%^12^. We were unable to test all PLWH due to few impediments. Some individuals reside far from the hospital, including those who live in other states, and some have their tests conducted in private facilities outside public health services. Only a small number were not tested due to being unreachable or refusing the test.

Despite these challenges, the response to prophylaxis with isoniazid (INH) and rifapentine (RPT) was promising, as all patients adhered to the treatment regimen. This suggests early intervention can effectively reduce the risk of progression to active tuberculosis. All 50 patients who were recommended for 3HP prophylaxis complied with the treatment protocol. However, note that we recorded three deaths due to tuberculosis before this scheme, highlighting the urgent need for healthcare providers to proactively identify potential high-risk cases within our population.

We observed that a three-month course of 3HP prophylaxis was as effective and safe in treating LTBI, with the advantage of being a shorter treatment duration in comparison to the previously standard nine-month INH regimen. Despite its lower cost, the TST is more widely available in developing countries due to its affordability and practicality. However, TST can be somewhat challenging for patients, as it requires a second visit to the health facility 48 to 72 h after the first visit. Additionally, lack of trained personnel to manage the subcutaneous injection and read the results on-site limits the broader use of TST compared to IGRA^17^. Nonetheless, IGRA does not require a follow-up visit, however, it also has its challenges. IGRA requires qualified personnel for proper execution and result validation, as well as laboratory infrastructure, and can yield indeterminate or inconclusive results, especially in individuals with low CD4 T-cell counts. Additionally, although the blood draw is simpler than managing a TST, it is just the first step of the process, and the test is more expensive^18^.

Three PLWH had indeterminate IGRA results. Notably, all indeterminate results involved patients with high T-CD4+ cell counts, a finding that does not align with other studies in which indeterminate IGRA was found among participants with low T-CD4 counts^19^, which was not the case in our study, may be associate with variables in the assay and other diseases. The association between low T-CD4+ cell counts and indeterminate IGRA results may stem from an insufficient immune response, leading to test bias, highlighting the potential limitations of IGRA due to severe immunosuppression.

## CONCLUSION

Finally, this strategy is being incorporated into the Brazilian National Health System (SUS) as an important measure for TB control and aims to become standard practice across the country. While it is successful in developed countries, it also shows promising results in countries with limited resources, such as Brazil. Considering Brazil has about 900,000 diagnosed PLWH^20^, we estimate nearly 140,000 cases of LTBI would need to be treated countrywide, given that 15% of PLWH who were tested with IGRA in our cohort were positive for LTBI. Prophylactic treatment could significantly impact the prevalence and incidence of TB infection in Brazil, with larger survival time.
